# Special Issue “Internet of Things for Smart Homes”

**DOI:** 10.3390/s19194173

**Published:** 2019-09-26

**Authors:** Ilsun You, Giovanni Pau, Valerio Mario Salerno, Vishal Sharma

**Affiliations:** 1 Department of Information Security Engineering, Soonchunhyang University, 22 Soonchunhyangro, Shinchangmyeon, Asan-si 31538, Korea; vishal_sharma2012@hotmail.com; 2 Faculty of Engineering and Architecture, Kore University of Enna, 94100 Enna, Italy; giovanni.pau@unikore.it (G.P.); valerio.salerno@unikore.it (V.M.S.)

**Keywords:** smart homes, information and communication technologies (ICT), internet of things (IoT), green communications, security and privacy, artificial intelligence, machine learning, wireless communications

## Abstract

Smart homes represent one of the principal points in the new ecosystem of the Internet of Things (IoT), both for the centrality of the home in the life of individuals and the significant potential concerning the diffusion of smart objects and innovative services. While IoT-oriented smart homes can revise how inhabitants interact with the domestic environment, each well-defined piece of technology necessitates precise network performance and distinct levels of security based on the sensitivity of the controlled system and the information it handles. This editorial presents a review of the papers accepted in the special issue. The issue has focused at obtaining high-quality papers aimed at solving well-known technical problems and challenges typical of IoT-oriented smart homes.

## 1. Introduction

In recent years, the progress of wireless protocols, the growth of cloud services, the refinement of low-energy and high-performance technologies, the practice of Artificial Intelligence, and other methods of convergence solutions based the Internet of Things (IoT) paradigm have launched a new era for smart homes. Technologies for IoT-oriented smart homes include sensors, interfaces, monitors, and several appliances, networked collectively to promote the automation and local/remote control of the domestic environment. The smart home represents a central hallmark in the new ecosystem of the Internet of Things, both for the centrality of the home in the life of every individual and for the enormous potential regarding the dissemination of objects and services. Thanks to the latest Information and Communication Technologies (ICT) and machine learning algorithms, the smart home environment can monitor the welfare and everyday life activities of residents, learning their distinct necessities and habits. The aim is to rearrange itself to them, thus improving their overall quality of life. Moreover, smart homes can skillfully manage the energy consumption of appliances and all other peculiarities related to the domestic environment, thus creating a healthier and energy-effective area for their inhabitants. While IoT-oriented smart homes can modify how inhabitants interact with the domestic environment, each distinct technology demands distinct levels of security based on the sensitivity of the controlled system and the information it handles. Smart Homes can be exposed to security threats and privacy breach that stem from current ICT and protocols.

This special issue has converged at collecting high-quality papers aimed at solving well-known technical problems and challenges typical of IoT-oriented smart homes. The primary purpose has been to combine innovative proposals efficiently, converging on the performance evaluation and the comparison with existing approaches. Among the 36 submissions, the guest editors picked 15 high-level contributions for publication after several rounds of reviews carried out by invited experts.

## 2. A Review of the Contributions in this Special Issue

Acknowledging that an extensive heterogeneity of use cases in various situations of the smart home with specific requirements can be realized, it is clear that no single wireless standard can satisfactorily predominate. There are several standards available on the market, developed over various frequency bands, and applying different communication protocols. Consequently, the selection of the best wireless connectivity technology for an IoT application for a smart home can be considerably challenging. The authors of [1] present a low-cost fog computing architecture for a home automation system that allows seamless communications among ZigBee and WiFi devices. The proposed approach is based on an open method that can be replicated and further validated. The suggested architecture is scalable and can include resource-constrained devices in the system that act as sensors or actuators. The results, regarding the latency, obtained in the performance evaluation, show that the fog computing approach can be harnessed for providing real-time or quasi-real-time responses. However, there is a conspicuous increment in current consumption that could be addressed by hardware manufacturers and software developers in the next generation of IoT fog computing applications. A self-powered, threshold-based wireless sensor is proposed in [2] to recognize unusual floor vibration situations. The authors develop a cantilever-type piezoelectric energy harvester to produce electric energy proportional to the amount of mechanical floor vibration. The prototype of the self-powered wireless sensor is produced, and its applicability is confirmed. A substantial relationship among floor impact sound, slab vibrations, and electric energy harvested is reported through the correlation study. Moreover, it is shown that harvested electric energy can be employed as a threshold to predict floor vibrations and the corresponding impact sound in buildings or apartments. The authors of [3] examine the performance of a dual-hop wireless/power line hybrid fading system employing an amplify-and-forward relay concerning outage probability and average bit error rate. Several mathematical methods are employed in the evaluation regarding the versatility and accuracy of the proposed method, and the influence of the hybrid fading channel and multidimensional impulse noise parameters. Some methods have promising and satisfactory results, while others could be further improved.

An energy management system for smart home refers to the employment of supervisory control and data retrieval, including the production, the delivery, and distribution of the electrical network. This theory has been broadly accepted to advise the future growth trend of power grids. Energy management deals with the real-time monitoring and arranging of numerous home appliances, based on user’s preferences via intelligent ambient systems controlled by a human-machine interface in smart houses, with the purpose of electricity cost minimization and energy use productivity enhancements. The authors of [4] propose a design plan for regulating a light system based on Arduino Uno microcontroller. The proposed system controls the lights based on nighttime and object detection. Meanwhile, it has capabilities to check the status of doors and to monitor objects. The hardware implementation of the suggested solution is carried with a specific prototype to validate the performance regarding simplicity, flexibility, reliability, and, mainly, the energy management. The obtained results are promising and suggest a large-scale implementation of the system proposed by the authors. The combination of several cloud energy management systems to accomplish advantages concerning the control of energy-efficient operations in smart buildings is introduced in [5]. The tests carried out in real scenarios demonstrate the improvements in both energy consumption and comfort conditions in a real pilot.

The expanding market for smart homes affords a more pleasant and more natural way of life to users while introducing new hurdles for protecting privacy. Moreover, due to the intrinsic features of wireless devices and smart appliances, such as resource limitations and the adoption of the wireless medium, they are expected to be exposed to different attacks. In such circumstances, cryptographic methods should be employed to preserve user privacy and wireless devices against various attacks. The authors of [6] analyze the security vulnerabilities in modern three-factor authentication and key agreement schemes. They introduce a system model for smart homes based on wireless devices in which a secure and lightweight three-factor authentication and key agreement scheme are employed. In the security verification, the authors prove that the proposed design satisfies the security specifications and resists against different attacks. The physical-layer security for a cognitive Internet of things model is assessed in [7]. The authors propose a cooperative jammer selection transmission protocol to preserve the information of primary users against eavesdropping. The numerical results reveal that the proposed protocol has more reliable primary secrecy performance than the non-security management model. The authors of [8] describes security threats, such as privacy infringement and personal information leak, in smart homes constituted by IoT devices. They address the evaluation method of these threats from the framework of situational awareness and exhibits the way of IoT threat knowledge from the viewpoint of decision-makers or managers. The obtained evaluations can provide support for a real-time response to the swiftly growing security operating environment of the future and can afford to periodic risk estimation in response to cyber threats, cyber-attacks, and cyber warfare from a perspective of national security. Software applications could manage smart home appliances. Consequently, the software protection of these devices is crucial. The authors of [9] introduce a reverse engineering analysis prevention method employing the relationship between Dalvik bytecode and machine code. The suggested scheme preserves the application from the static analysis by restricting the exposure of the Dalvik bytecode, which is easier to investigate confronted with machine code. The reverse engineering defense of the scheme presented by the authors is confirmed through reverse engineering analysis tests on its application.

Smart home environments represent a significant interdisciplinary research field of computer science that is situated at the crossing of computer networks, applied computing, and embedded systems. In detail, the center on smart features led the research community to the application and experimentation of different intelligent computing methods and networks for improving the performance of smart home environments. The authors of [10] introduce a design project for a single-equipment with a multiple-application system for commanding a robot car based on Arduino in a smart environment. The suggested system practices a mechanism for managing the robot car based on hand gesture recognition and commands it based on a mobile application with touch buttons and voice recognition. The results obtained in extensive experiments confirm the simplicity, adaptability, dependability, specificity, and real low cost of the proposed system. Consequently, it could be easily implemented in smart cars or smart homes. Knowledge of things framework, able to share local information between IoT devices requiring similar or identical data at the edge, is proposed in [11]. The prototype developed by the authors consists of a smart mirror and a smart doorbell which require the same knowledge for face recognition. The outcomes of the investigations reveal that the proposed framework decreases both the response time to practice intelligent IoT devices and the power consumption for computation. The authors of [12] suggest a novel decision-making practice able to optimize the cruise cabin comfort. The proposed solution leverages an ontology-based description of the passengers, their health condition, indoor comfort indicators, and sensors and actuators to fit made-to-measure comfort adaptations to them. The obtained results reveal a high approval rate of automatic comfort optimization based on holistic comfort. The authors of [13] aim at developing a smart platform for assisting elderly and non-self-sufficient people in smart homes, to let them feel as comfortable as possible. The proposed system, composed of several smart objects to be incorporated into everyday life, is tested on both final users, i.e., self-sufficient and non-self-sufficient seniors, and caregivers, and the assessment is reasonably satisfactory. A smart solution, based on wirelessly interconnected sensors, for proper wellness ascertainment of older adults, living alone in smart homes, is proposed in [14]. This system strives to afford healthcare monitoring of older people, along with the main aim of higher wellness measurement classification accuracy and precision for better healthcare. The obtained results reveal that the proposed solution outperformed the compared techniques over the dataset in terms of accuracy and precision. The authors of [15] suggest a new configuration algorithm for endpoints in smart homes intending to let them operate most efficiently according to several parameters obtained by the monitored environment. The recommended approach performs predictions applying a mathematical model, where all connected costs in the information reception and consumption are identified and quantified. Empirical validations are carried out practicing several simulation scenarios and real deployments, and the acquired results confirm the excellent performance of the proposed algorithm and a proper efficiency improvement system operation compared to other methods.

## References

[1] Froiz-Maguez I., Ferna¡ndez-Caramas T.M., Fraga-Lamas P., Castedo L. (2018). Design, Implementation and Practical Evaluation of an IoT Home Automation System for Fog Computing Applications Based on MQTT and ZigBee-WiFi Sensor Nodes. Sensors.

[2] Jung B.C., Huh Y.C., Park J.W. (2018). A Self-Powered, Threshold-Based Wireless Sensor for the Detection of Floor Vibrations. Sensors.

[3] Chen Z., Ye C., Yuan J., Han D. (2019). MGF-Based Mutual Approximation of Hybrid Fading: Performance of Wireless/Power Line Relaying Communication for IoT. Sensors.

[4] Mumtaz Z., Ullah S., Ilyas Z., Aslam N., Iqbal S., Liu S., Meo J.A., Madni H.A. (2018). An Automation System for Controlling Streetlights and Monitoring Objects Using Arduino. Sensors.

[5] Marin-Perez R., Michailidis I.T., Garcia-Carrillo D., Korkas C.D., Kosmatopoulos E.B., Skarmeta A. (2019). PLUG-N-HARVEST Architecture for Secure and Intelligent Management of Near-Zero Energy Buildings. Sensors.

[6] Shin S., Kwon T. (2019). A Lightweight Three-Factor Authentication and Key Agreement Scheme in Wireless Sensor Networks for Smart Homes. Sensors.

[7] Xie P., Xing L., Wu H., Seo J.T., You I. (2018). Cooperative Jammer Selection for Secrecy Improvement in Cognitive Internet of Things. Sensors.

[8] Park M., Oh H., Lee K. (2019). Security Risk Measurement for Information Leakage in IoT-Based Smart Homes from a Situational Awareness Perspective. Sensors.

[9] Na G., Lim J., Lee S., Yi J.H. (2019). Mobile Code Anti-Reversing Scheme Based on Bytecode Trapping in ART. Sensors.

[10] Ullah S., Mumtaz Z., Liu S., Abubaqr M., Mahboob A., Madni H.A. (2019). Single-Equipment with Multiple-Application for an Automated Robot-Car Control System. Sensors.

[11] Jang I., Lee D., Choi J., Son Y. (2019). An Approach to Share Self-Taught Knowledge between Home IoT Devices at the Edge. Sensors.

[12] Nolich M., Spoladore D., Carciotti S., Buqi R., Sacco M. (2019). Cabin as a Home: A Novel Comfort Optimization Framework for IoT Equipped Smart Environments and Applications on Cruise Ships. Sensors.

[13] Borelli E., Paolini G., Antoniazzi F., Barbiroli M., Benassi F., Chesani F., Chiari L., Fantini M., Fuschini F., Galassi A. (2019). HABITAT: An IoT Solution for Independent Elderly. Sensors.

[14] Ujager F.S., Mahmood A. (2019). A Context-Aware Accurate Wellness Determination (CAAWD) Model for Elderly People Using Lazy Associative Classification. Sensors.

[15] Sanchez-de Rivera D., Bordel B., Alcarria R., Robles T. (2019). Enabling Efficient Communications with Resource Constrained Information Endpoints in Smart Homes. Sensors.

